# VICTIMIZATION EXPERIENCES AND COGNITIVE HEALTH: VARIATION BY SEXUAL MINORITY STATUS

**DOI:** 10.1093/geroni/igae098.0095

**Published:** 2024-12-31

**Authors:** Harry Barbee

**Affiliations:** Johns Hopkins Bloomberg School of Public Health, Baltimore, Maryland, United States

## Abstract

Studies have revealed how experiences of victimization generate stress and harm cognitive health outcomes. For minority populations, such as sexual minorities, victimization experiences may occur more frequently, thus posing a greater risk to their cognitive health outcomes in later life stages. This project explores this possibility by analyzing how different types of victimization experiences, including being treated with less respect, receiving poorer services, and being threatened or harassed influence cognitive health outcomes among older adults in the United States. To achieve this goal, I examined data from the Health and Retirement Study, a nationally representative survey of middle-age and older adults in the United States. Specifically, I employed adjusted logistic regression models and adjusted Poisson regression models to test the extent to which experiencing different forms of victimization influenced subjective cognitive health, via self-rated memory, as well as a variety of objective markers of cognitive health. All models controlled for key sociodemographic variables. Preliminary results indicate that respondents who experienced different types of victimization also reported significantly worse cognitive health outcomes. Additionally, sexual minorities who reported victimization experiences had worse cognitive health outcomes than the heterosexual respondents who also reported victimization experiences. Together, these results demonstrate how perceived experiences of victimization can harm cognitive health, and also reveals potential explanations for why older sexual minorities face greater risk of cognitive impairment than their heterosexual counterparts.

